# Conifer Green Needle Complex in Patients with Precancerous Gastric Lesions: An Observational Pilot Study

**DOI:** 10.1155/2016/3848409

**Published:** 2016-11-28

**Authors:** Vladimir Bespalov, Alexander Sherbakov, Viktor Novik, Valentin Kalinovsky, Kamran Shamsi, Vagif Soultanov

**Affiliations:** ^1^Laboratory of Cancer Chemoprevention and Oncopharmacology, N. N. Petrov Research Institute of Oncology, Ministry of Healthcare and Social Development, Saint Petersburg, Russia; ^2^Department of Endoscopy, N. N. Petrov Research Institute of Oncology, Ministry of Healthcare and Social Development, Saint Petersburg, Russia; ^3^Laboratory of Pathological Morphology, N. N. Petrov Research Institute of Oncology, Ministry of Healthcare and Social Development, Saint Petersburg, Russia; ^4^Laboratory of Biochemistry, N. N. Petrov Research Institute of Oncology, Ministry of Healthcare and Social Development, Saint Petersburg, Russia; ^5^Solagran Limited, Biotechnology Company, 98-106 Moray St., South Melbourne, VIC 3205, Australia; ^6^Saint Petersburg State Forest Technical Academy, Saint Petersburg, Russia

## Abstract

*Objectives*.* Helicobacter pylori* infection is common and can lead to precancerous gastric lesions. Standard antibiotic therapy has a failure rate of more than 25% from antibiotic resistance. The primary aim of this observational pilot study was to test the feasibility of a large-scale clinical trial of Conifer Green Needle Complex (CGNC) to treat precancerous gastric lesions. Secondary aims were to investigate* H. pylori* infection, stomach function, and histopathology of the gastric mucosa.* Methods*. A tablet form of CGNC (extracted from* Pinus sylvestris* and* Picea abies* (L) Karst) was prescribed to 26 patients with precancerous gastric lesions (two tablets, 100 mg CGNC/tablet, three times per day for six months). Another 24 patients received no treatment.* Results.* Compared with control patients, CGNC-treated patients showed total or partial regression (using the quantitative Rome III diagnostic criteria) of dyspeptic symptoms (92.3%, *p* < 0.0001), eradication of* H. pylori* infection (57.1%, *p* < 0.03), a reduction in endoscopic signs of gastritis (92.3%, *p* < 0.001), an increase of pepsinogen-pepsin in the gastric juice (57.7%, *p* < 0.05), and total regression or reduction in the degree of intestinal metaplasia (46.2%, *p* < 0.05) and lymphoplasmacytic infiltration (53.8%, *p* < 0.05).* Conclusions.* This study justifies a randomised-controlled trial with CGNC in patients with atrophic gastritis.

## 1. Introduction

Stomach cancer is still the fourth most common cancer and the second most common cause of cancer-related deaths. For all primary stomach cancer patients, the five-year survival rate varies between 8.4 and 32.1% depending on the country, and in most countries it does not exceed 30% [1].

Although the aetiology of stomach cancer is thought to be multifactorial,* Helicobacter pylori* infection is the most important risk factor [2, 3] and World Health Organization has classified* H. pylori* as a Class I carcinogen for gastric cancer [4]. Numerous studies have established the clear connection between* H. pylori* infection and the development of gastric adenocarcinoma and lymphoma of the gastric mucosa [5, 6].* H. pylori* infects half of the world's population.* H. pylori* colonisation causes inflammation of the gastric mucosa leading to gastric precancerous lesions such as atrophy, intestinal metaplasia, and dysplasia [7]. Clinical symptoms of disease appear only in 10–20% of those infected. These diseases include peptic ulcers of the duodenum and stomach, acute gastritis, chronic nonatrophic and atrophic gastritis, adenocarcinoma, and B-cellular gastric lymphoma [8].

Factors that lead to the regression of precancerous gastric lesions break the cascade of gastric carcinogenesis and can serve as an effective measure for prevention of cancer. However, total regression of intestinal metaplasia is impossible to be guaranteed because the mucosa is subjected to sampling errors when selecting sites for biopsy. Therefore, the quantitative evaluation of the risk of stomach cancer in patients with precancerous lesions in the gastric mucosa is hard to evaluate. In recent times, noninvasive and serological diagnostic markers of* H. pylori* and atrophic gastritis have been developed [9]. However, the direct diagnosis of* H. pylori*, atrophic gastritis, intestinal metaplasia, and dysplasia (by morphological analysis of biopsy material obtained during gastroscopy) is widely used and was the main diagnostic tool in this trial.

Eradicating* H. pylori* and thus reducing risk of gastric cancer have become more difficult. Because of antibiotic resistance, standard antibiotic therapy does not eradicate* H. pylori* eradication in more than 25% of people [8]. For this reason, there is increasing interest in other treatment options, including phytotherapies [10].

Conifer needle extract has been used for decades in Russia for its antibacterial, antifungal, antiviral, and anti-inflammatory activity. In 2000, the composition of a product called Coniferous Chlorophyll Carotene Paste (CCCP) was controlled by a Russian State Standard (GOST). The components of CCCP include chlorophyll derivatives, carotenoids, phytosterols, polyprenols, and vitamins E and K1 and other compounds [11]. A more advanced and pure isolate with a highly controlled composition is now available and is known by the TGA Australian Approved Name (AAN) Conifer Green Needle Complex (CGNC) and Bioeffective® A.

CGNC is a unique complex with antioxidant and antibacterial activity. Antibacterial and antifungal activities along with antioxidant activities are thought to contribute to anticancer activity [12]. The fact that CGNC has all of these activities contributes to its therapeutic effect. There is a range of evidence suggesting that components of CGNC might be associated with reducing the risk of cancer, including evidence for chlorophyll derivatives [7, 13–16], carotenoids [17–19], phytosterols [20–22], squalene [23], and vitamin E [24]. Vitamin K1, a component of CGNC, might have a role in decreasing the risk of hepatocellular cancer [25], although the role of vitamin K2 remains unclear [26, 27].

While the effectiveness of the components of CGNC is important, it is the synergistic effects of the complex that is of interest for this study. CGNC has antimicrobial activity, suppressing* H. pylori* in vitro [28, 29], as well as 83 other strains of bacteria and 16 strains of* Candida* [30]. The strong antioxidant activity of CGNC contributes to its effectiveness as a hepatoprotector in rats in a model of liver damage with carbon tetrachloride [31]. CCCP could also be effective in reducing the risk of malignant disease [32]. In addition, in patients with atrophic gastritis, CCCP increased the production of hydrochloric acid and pepsin-pepsinogen in stomach mucosa, as well as improving endoscopic signs of precancerous changes [33].

In light of these previous studies, this study is a concurrent examination of signs of atrophic gastritis and infection with* H. pylori* in patients. The effect of CGNC on* H. pylori* in stomach mucosa was examined as well as the clinical and endoscopic signs of gastritis, stomach function, atrophy, intestinal metaplasia, and dysplasia of gastric mucosa in patients with precancerous gastric lesions. The results suggest that it is important to conduct a larger randomised-controlled trial with CGNC in patients with atrophic gastritis.

## 2. Materials and Methods

### 2.1. Test Substance

CCCP was originally prepared and characterised by F. T. Solodky and A. L. Agranat at the Leningrad Forest Technical Academy in the 1930s. The biologically active nutritional additive (BANA), Lesmin, was manufactured by Fitolon Ltd., St. Petersburg, Russia. Lesmin tablets contain the equivalent of 100 mg CGNC on a dry basis per tablet, a formulation which has a lower concentration than the TGA-approved Bioeffective A (320 mg CGNC) in Australia.

CGNC is prepared as follows. Freshly collected, small branches of* Pinus sylvestris* and* Picea abies* (L) Karst, in the weight ratio of 55 : 45, were extracted using a hydrocarbon solvent. Concentrated dewaxed extract, free of hydrocarbon and light essential oils, was saponified using diluted alkali with further water dilution to pH 8-9. The resulting substance is a dark green viscous paste comprising several hundred components called CGNC. CGNC of Bioeffective A is an Australian TGA-approved therapeutic substance for oral and topical application as a strong antioxidant with an oxygen radical absorbance capacity (ORAC 5.0) as determined at the Brunswick Laboratories (USA) [34].

The primary components present are resin acids (ca. 20% w/w) comprising mainly bicyclic and tricyclic diterpene acids, terpenoid alcohols (ca. 19% w/w), higher fatty acids (HFA) (ca. 10% w/w), esters of higher fatty acids (ca. 9% w/w), and polyprenols (ca. 1.5% w/w). Other significant components include phytosterols (ca. 0.9% w/w), carotenoids (ca. 0.4% w/w), chlorophyll derivatives (ca. 0.6–1.2% w/w), vitamin E acetate (ca. 127 *μ*g/g), and vitamin K1 (ca. 12 *μ*g/g). The balance of CGNC is 40% water. The composition of CGNC is controlled by an Australian TGA Draft Compositional Guideline.

### 2.2. Recruitment of Subjects

The study was approved by the Ethics Committee of the N. N. Petrov Research Institute of Oncology. The research in humans followed the guidelines of the Declaration of Helsinki 1975, revised Tokyo 1989.

Recruitment of patients was conducted at the oncology hospital of the N. N. Petrov Research Institute of Oncology between 2007 and 2009. The patients were admitted to the hospital for examination of potential stomach cancer. Those patients were considered for the study if a diagnosis of stomach cancer was excluded by fibrogastroscopy but symptoms of chronic atrophic gastritis were found. Patients were included in the trial if they satisfied the inclusion criteria and gave written informed consent after having the requirements of the trial and the consent process discussed with them during consultation with a clinician.

**Figure 1:**
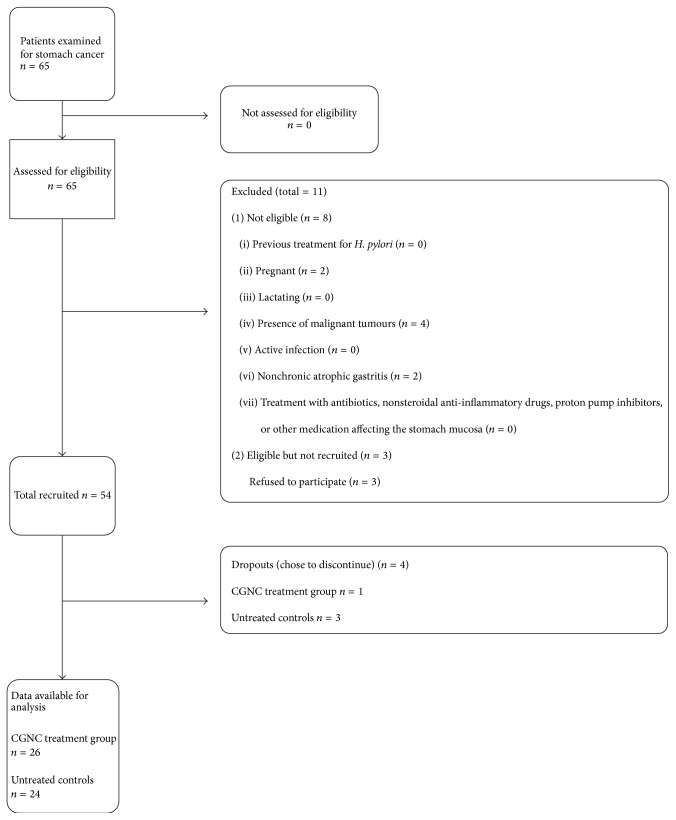
Flow diagram showing the number of individuals at each stage of the study. CGNC: Conifer Green Needle Complex.

Patients included were men and women aged 40 to 65 years who had complaints of dyspepsia symptoms, endoscopic signs of chronic gastritis with atrophy of the gastric mucosa, and a diagnosis of chronic atrophic gastritis proven by histology. Patients who previously had treatment for* H. pylori* were excluded from the trial. Patients were also excluded from the trial if they were pregnant and lactating or had malignant tumours, active infection, or fever or were currently receiving antibiotics, nonsteroidal anti-inflammatory drugs, proton pump inhibitors, or other medications affecting the stomach mucosa. The number of patients involved in each stage of the recruitment is shown in a flow diagram (Figure 1), as suggested by the STROBE guidelines [35].

According to the practice adopted at N. N. Petrov Research Institute of Oncology, patients diagnosed with atrophic gastritis and other precancerous changes are subject to patient care to ensure timely detection of the development of gastric cancer. For this study, we compared the CGNC treatment with the true clinical alternative (usual care). For this group of patients, “no treatment” was the clinical alternative. Using “no treatment” as the control allowed us to observe both the specific and nonspecific effects of the CGNC. All recruited patients had no indications for treatment; thus no treatment in control group is ethically justified. The authors understand that there is debate around the use of placebos [36, 37]; however, if this observational study indicates the feasibility for a randomised-controlled trial, placebos would be integrated into the future study design.

Patients in both groups had an appointment with a doctor once a month to assess their condition; the doctor asked questions about patients' diet and other lifestyle factors to ensure that these had not changed the trial.

### 2.3. Treatment of Subjects

Patients received 600 mg per day of CGNC (two Lesmin tablets, three times per day) before food for six months. The control group received no treatment at all. In the treatment group, the patients received treatment for one month and then attended an appointment where they answered a questionnaire about the compliance with the treatment regimen. The protocol called for patients who were not compliant with treatment regimens to be excluded from the trial, although this did not occur. However, dropouts did occur, with one patient from the treatment group and three patients from the control group dropping out of their own accord and not completing the trial.

Fifty-four patients participated in this study, with 27 patients in each group. One group of patients was assigned to take CGNC therapy. The other was the control group, where patients did not receive any treatment. The trial continued for six months and after the four patient dropouts, the 50 remaining patients attended all the scheduled examinations. Thus, the CGNC treatment group consisted of 26 patients, whereas the untreated control group consisted of 24 patients.

### 2.4. Safety Monitoring

Side effects were evaluated using a questionnaire filled in by the medical practitioner during consultations with the patients at each monthly visit. The questions were mainly based on discussion of any symptoms of the gastrointestinal tract, the urinary system, the reproductive organs, the nervous system, the skin, and any other symptoms not covered by the specific questions.

### 2.5. Clinical Evaluation of Subjects

All the examinations listed below were conducted in patients before the commencement of the therapy or the monitoring period (in the control group) and also at 6 months (at completion of the trial).

A quantitative evaluation scale with four grades (no symptoms, 1st degree symptoms, 2nd degree symptoms, and 3rd degree symptoms) was developed for each examination based on the standard Rome III diagnostic criteria for functional gastrointestinal disorders. Analysis of clinical symptoms of dyspepsia included evaluation of pain in the stomach area, the feeling of heaviness in the epigastric area, nausea, eructation, and other pathological symptoms of the gastrointestinal tract. The symptoms were given one of four grades, which were then allocated points from one to five on a Likert scale. A grade of no symptoms was given one point on the Likert scale; a grade of 1st degree symptoms (infrequent/intermittent symptoms, less than once per week, which were weak to moderate and short-lasting) was given two points; a grade of 2nd degree symptoms (periodic symptoms, at least once per week but not every day, which were moderate, weak, and short-term) was given three points; a grade of 3rd degree symptoms (frequent symptoms, almost every day, which were moderately severe and prolonged) was given four points; a grade of 3rd degree symptoms (where symptoms caused extreme discomfort and disturbed daily activities and sleep and required the patient to rest) was given a score of five points.

### 2.6. Fibrogastroscopy

Examination was carried out using a video endoscope manufactured by Olympus (Japan). All patients had the endoscopic examination in the morning on an empty stomach. During gastroscopy of the patients, the following samples were taken: gastric juice, not less than 4 biopsy samples from the antral section and not less than two biopsy samples from the corner, and body of the stomach. In cases where lesions were identified in other sections of the stomach, biopsy samples were taken from those sections as well.

The endoscopic pattern of the stomach mucosa was evaluated using the following criteria (hyperaemia, oedema, thinning, and granularity): (i) light hyperaemia in the form of individual small foci in a single section (1st degree), moderate focal hyperaemia predominantly located in a single section (2nd degree), and intensive hyperaemia evenly spread in all sections of the stomach (3rd degree); (ii) light oedema predominantly in a single section (1st degree), moderate oedema predominantly in one or two sections (2nd degree), and marked oedema in all sections of the stomach (3rd degree); (iii) light thinning predominantly in the antral section with weakened peristalsis and a visible vascular pattern in separate sections of the mucosa (1st degree), moderate thinning in the antral section with a visible vascular pattern and sluggish peristalsis and some flatness of the folds in the body of the stomach (2nd degree), and manifest thinning in the antral section with a clear vascular pattern and no peristaltic waves, flatness of folds and sluggish peristalsis in the stomach body (3rd degree); (iv) weak granularity predominantly in the antral section in the form of small flat papules (1-2 mm in diameter) located in separate sections of the mucosa (1st degree), moderate granularity predominantly in the antral section in a form of papules of 2-3 mm in diameter and 3 mm tall (2nd degree), and expressed granularity of the mucosa in the antral and/or other sections in a form of “cobble-stone pavement” (3rd degree).

### 2.7. Analysis of Functional Activity of the Stomach

The pH of the gastric juice was measured at a dilution of 1 : 10, using a pH-meter and standard calibrating buffer solutions. Biochemical analysis of pepsinogen-pepsin in the mucosa and gastric juice was carried out using photofilms, as described in our earlier publication [38].

### 2.8. Cytological Evaluation

Material for cytological analysis was obtained during fibrogastroscopy using samples from gastric mucosa, sampled during biopsy. After fixation in 96% alcohol, two smears were stained with haematoxylin and eosin. The presence of intestinal metaplasia cells, dysplasia cells, and lymphocytes and plasma cells was evaluated during cytological analysis as per the following classification: in single cells (1st degree), in single layers of cells (2nd degree), and in many layers of cells (3rd degree).

### 2.9. Histological Evaluation

Samples of gastric mucosal tissue, taken during biopsy, were stained with haematoxylin and eosin. Histological analysis was used for evaluation of the degree and type of intestinal metaplasia (complete or incomplete), the degree of neutrophilic (lymphoplasmacytic) infiltration, and the degree of atrophy according to the updated Sydney classification and gradation of gastritis [39] with identification of four degrees (0, none; 1, mild; 2, moderate; and 3, marked). Intestinal metaplasia was classified as follows: mild (1st degree), in cases of a few individual foci of metaplasia in the preparation; moderate (2nd degree), in cases of group foci of metaplasia; and marked (3rd degree), in cases of a predominance of foci of metaplasia or total metaplasia. Lymphoplasmacytic infiltration was classified as follows: mild (1st degree), individual evenly distributed lymphocytes and plasma cells in the mucosal layer; moderate (2nd degree), homogeneously friable infiltration of a mucosal layer by lymphocytes and plasma cells; and marked (3rd degree), dense infiltration of a mucosal layer by lymphocytes and plasma cells. Gastric mucosal atrophy was classified as mild, moderate, and marked, using a visual-analogue scale [39].

Dysplasia was classified as follows: mild dysplasia (1st degree), extension of pits, increased diameter and hyperchromatosis of nuclei, an increase of nuclear-cytoplasmic ratios, glands that are partially overlaid by adenomatous epithelium, and rare multirowed structure; moderate dysplasia (2nd degree), marked extension of pits, increased diameter and hyperchromatosis of nuclei, increased nuclear-cytoplasmic ratios, glands that are often overlaid by adenomatous epithelium, frequent multirowed structure; and marked dysplasia (3rd degree), the outer side of the glands that is overlaid by basophilic columnar cells with prolonged nuclei, presence of goblet cells, in some cases papillomatous outgrowth of epithelium, on the inner side, pyloric glands or intestinal crypts, cellular atypia, anisokaryosis, nuclear hyperchromatosis, sharp increase of nuclear-cytoplasmic ratios, and widespread pseudostratification.

### 2.10. Determination of* H. pylori* Infection

During gastroscopy and immediately after obtaining biopsy samples with one piece of tissue from the antral section, a rapid urease test for detecting* H. pylori* was carried out using* Helicobacter pylori* test kits (HelPyl test) manufactured at Ama Ltd., St. Petersburg, Russia. The HelPyl test strip was placed on microscope slides and the biopsy material was placed onto the test strip's surface. The time of visible colour change from yellow to blue in the contact zone with the biopsy material was registered. In cases when the colour shift took less than three minutes, the test was considered as positive. For cytological analysis, two smears of biopsy material from the antral section of the stomach were fixed in 96% alcohol and then stained by azor-eosin as per the Leishman method for detection of* H. pylori*.* H. pylori* was also detected during histological analysis of biopsy material from the antral section of the stomach. A patient was considered to be infected if the quick urease test was confirmed by detection of* H. pylori* by histological and/or cytological analysis of biopsy material from the antral section of the stomach.

### 2.11. Statistical Analysis

Given that this is a pilot feasibility study for effectiveness of the treatment, the strategy for allocation of subjects was paired selection of patients (sex- and age-matched, ±3 years) who had similar symptoms of dyspepsia and endoscopic signs of chronic atrophic gastritis, followed by allocation to groups using random sampling. Matching is a valid approach that can help reduce confounding bias in observational studies [40]. The random sampling procedure was conducted using a table of random numbers generated by the Statistica program. Although this type of matching followed by random sampling cannot take into account all confounding factors, it can be particularly useful with small sample sizes.

Statistical analysis of the results was conducted using *χ* ^2^ criterion and Fisher's exact test, with significance set at *p* < 0.05.

## 3. Results

### 3.1. Characteristics of Patients Prior to Treatment

All patients had baseline clinical symptoms of dyspepsia. Most often, the patients had several symptoms such as pain in the gastric area, a feeling of heaviness in the epigastric area, nausea, eructation, flatulence, and bowel disorders. In all examined patients, gastroscopy revealed symptoms of chronic atrophic gastritis with atrophy predominantly in the antral section of the stomach (Table 1). In all patients, atrophy of the gastric mucosa was confirmed by histological analysis. In most of the cases, patients were diagnosed with a mild-to-moderate degree of atrophy and only a few patients had the marked degree. Adenomatous polyps of the stomach mucosa were found in 26.9% of the patients selected for experimental group and 29.2% of the patients in the control group.* H. pylori* infection was registered in 53.8% and 54.2%, respectively. Mild or moderate dysplasia was found in 11.5% of the patients selected in the CGNC treatment group and 12.5% of the patients in the control group (Table 1).

Stomach function was analysed based on the production of hydrochloric acid (normal pH of the gastric juice is 1.6–1.8) and pepsinogen-pepsin levels (the normal level of pepsinogen-pepsin in the gastric juice and stomach mucosa is 100–120 mg/100 mL and 100–120 mg/g of tissue, resp.) [38]. In all patients, stomach function was decreased compared with healthy parameters (the normal range) as exhibited by an increase in the pH of the gastric juice and/or a reduced level of pepsinogen-pepsin in the gastric juice and/or gastric mucosa.

Histological and cytological analysis showed intestinal metaplasia of the 1st, 2nd, or 3rd degree in 61.5% and 70.8% of the patients, respectively, from the above-mentioned groups. In almost all cases, intestinal metaplasia was complete and it was incomplete in only three cases.

### 3.2. Symptoms of Dyspepsia and Endoscopic Signs of Gastritis After Treatment

A six-month course of CGNC therapy resulted in the complete regression or significant reduction of symptoms of dyspepsia in 92.3% of the patients compared with 12.5% of untreated controls (Table 2, *p* = 0.00000, Fisher's exact test). If patients had not shown any symptoms of pathological dyspepsia for at least one last month, they were considered to have complete regression of dyspepsia symptoms. If the intensity of dyspepsia symptoms weakened or if some of the symptoms disappeared altogether for at least one month, then the patients were considered to have a partial regression of dyspepsia symptoms. There were no changes observed in 7.7% of CGNC-treated patients compared with 58.3% of untreated controls (Table 2, *p* = 0.00013, Fisher's exact test). There were 0% of CGNC-treated patients with worsening of symptoms compared with 29.2% of untreated controls (Table 2, *p* = 0.00347, Fisher's exact test). Before the CGNC treatment, bowel disorders were recorded in 18 patients (14 patients with constipation and 4 patients with diarrhoea). CGNC therapy resulted in normalisation of bowel activity in 17 of the 18 patients.

After CGNC therapy, the improvement of the endoscopic pattern of the stomach mucosa (reduction of hyperaemia, oedema, thinning, and granulation of the stomach mucosa) was observed in 92.3% of the patients, no changes were found in 7.7%, and no worsening of the condition was found in any patient. At the same time, in the control group, these parameters were 16.7%, 66.7%, and 16.7%, respectively. Thus, for these parameters, the differences between the treatment and control groups were statistically significant (Table 2, *p* = 0.00000, 0.00001, and 0.04614, respectively, Fisher's exact test).

**Figure 2:**
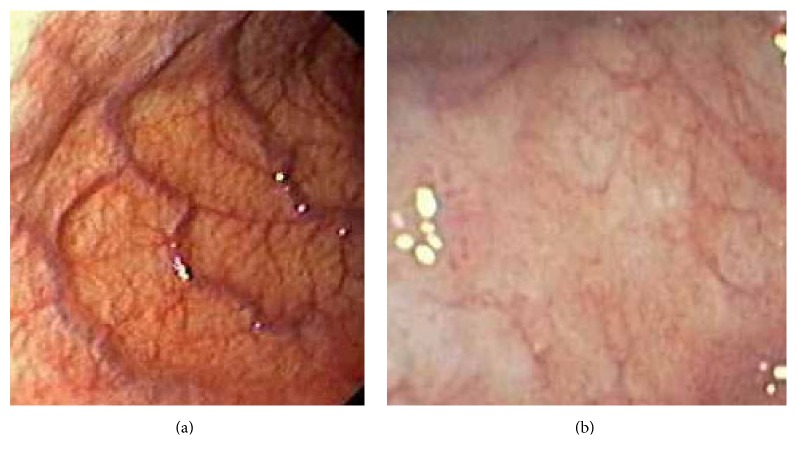
Typical examples of endoscopic observational patterns of atrophic gastritis in the antral section of the stomach in patients before (a) and after (b) treatment with Conifer Green Needle Complex.

Before treatment with CGNC, endoscopic observation of atrophic gastritis in the antral section of the stomach showed a pattern of hyperaemia and mucosal thinning (Figure 2(a) shows a representative example). After treatment with CGNC, regression of hyperaemia and improvement of mucosal thinning could be seen (Figure 2(b) shows a representative example).

### 3.3. *H. pylori* Infection

While this pilot study's primary aim was to examine precancerous gastric lesions, a preliminary examination of* H. pylori* infection in the patients with gastric lesions was also performed. Prior to treatment, 14 patients from the treatment group and 13 patients from the control group were identified with* H. pylori* infection (Table 3). After six months of CGNC therapy (at this lower concentration of CGNC),* H. pylori* infection could no longer be observed in 8 of the 14 (57.1%) infected patients compared with two of the 13 (15.4%) infected patients in the control, untreated group (Table 3, *p* = 0.0277, Fisher's exact test).

### 3.4. Functional Activity of the Stomach After CGNC Treatment

CGNC treatment improved the functional activity of the stomach in the patients (Table 4). As a result of six months of therapy with CGNC, the pH of gastric juice decreased back towards normal levels (improved) in 30.8% of the patients, whereas a decrease in the pH of gastric juice was only observed in 16.7% of patients in the control group. However, this difference was not statistically significant. On the other hand, in the CGNC group following treatment, an increase in the pH of gastric juice (i.e., worsening) was found only in 15.4% of the patients, whereas in the control group an increase in the pH gastric juice was found in 45.8% of patients. This difference was statistically different (Table 4, *p* = 0.01658, Fisher's exact test).

After CGNC therapy the activity of pepsinogen-pepsin in the gastric juice increased (improvement) in 57.7% of the patients, whereas in the control group 29.2% of patients had an increase in activity. This difference was statistically significant (Table 4, *χ* ^2^ = 4.12140, *p* < 0.05). Moreover, in eight (30.8%) patients, the activity of pepsinogen-pepsin in the gastric juice was restored to normal levels as a result of the CGNC therapy. After six months, there was no statistically significant difference in changes of the pepsinogen-pepsin level in the stomach mucosa between the experimental group and control group.

### 3.5. Morphological Features of Gastritis, Atrophy, and Precancerous Lesions

CGNC treatment reduced the degree of intestinal metaplasia and lymphoplasmacytic infiltration in patients (Table 5). Histological and/or cytological analysis of the biopsy samples of the gastric mucosa revealed that the CGNC therapy resulted in a decrease of the degree of intestinal metaplasia (or its total regression in samples taken) in 46.2% of patients compared with a decrease in only 16.7% of control, untreated patients (*p* = 0.02085, Fisher's exact test). This difference is statistically significant. Histological and/or cytological analysis of biopsy samples from gastric mucosa also demonstrated that CGNC therapy led to a reduction of the degree of lymphoplasmacytic infiltration in 53.8% of the patients compared with only 20.8% in control, untreated patients (*χ* ^2^ = 5.77304, *p* < 0.05). This difference is statistically significant.

**Figure 3:**
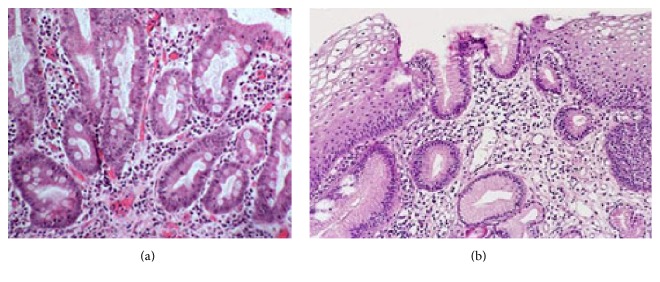
Typical examples of histological analysis of biopsy sample from the antral section of the stomach showed intestinal metaplasia before treatment with Conifer Green Needle Complex (a) and total regression of intestinal metaplasia after treatment (b).

A typical example of histology shows complete metaplasia in the antral section of the stomach during atrophic gastritis before treatment with CGNC (Figure 3(a)). After treatment with CGNC, regression of metaplasia was typically observed (Figure 3(b)).

After six months, there were no significant changes in the parameters of atrophy or dysplasia of gastric mucosa either in the CGNC group or in the control group. No significant differences between the groups were registered.

### 3.6. Assessment of Side Effects of CGNC

All patients tolerated CGNC well. There were no cases of any clinically significant toxic or adverse reactions.

## 4. Discussion

Use of CGNC in patients with chronic atrophic gastritis resulted in significant (in comparison with the control patients) regression of dyspeptic symptoms, endoscopic signs of chronic gastritis, and* H. pylori* infection; decreased pH and increased pepsinogen-pepsin level in gastric juice; and decreased the degree of intestinal metaplasia and lymphoplasmacytic infiltration in gastric mucosa.

No improvement, or worsening, of these parameters was found in most patients from the control group over the six months. However, improvement of intestinal metaplasia and histologic gastritis was observed in a small number of patients in the control group after six months of observation. This is most likely due to the natural course of chronic gastritis, where some patients can have spontaneous short-term improvement as shown in histologic assessment of intestinal metaplasia and gastritis.

The six-month course of CGNC therapy did not have any significant effect on the histological parameters of atrophy. Despite the marked therapeutic effects of CGNC on the clinical symptoms of dyspepsia, the endoscopic signs of gastritis, stomach function and inflammation of the gastric mucosa in patients with chronic atrophic gastritis, and the degree of atrophy of gastric mucosa did not significantly change. This suggests perhaps that morphological restructuring of the gastric mucosa, in terms of the reduction of atrophy, requires a longer course of treatment, although this will require further investigation during a larger study.

Therapeutic activity of CGNC in patients with atrophic gastritis is related to the activity of chlorophyll derivatives, carotenoids, vitamin E, phytosterols, and other substances included into the so-called phytoncide conifer complex [41–46]. Chlorophyll and phytoncidal complex (neutral components, free acids especially resin acids, and volatile oils) have antibacterial and antifungal activity that likely explains the capacity of CGNC to eradicate* H. pylori* infection. Carotenoids stimulate immune reactions against* H. pylori* [47]. In patients with chronic gastritis and* H. pylori* infection, the content of *β*-carotene in gastric juice was significantly lower than that in uninfected patients [48]. Meta-analysis of 51 studies of the effect of eradication of* H. pylori* on chronic gastritis was conducted, leading to the conclusion that such eradication suppresses the activity of gastritis and inflammation in practically all patients and in some patients such suppression has a positive effect on atrophy and intestinal metaplasia [49].

Recently, due to an increase in antibiotic resistance, standard antibiotics do not clear* H. pylori* in more than 25% of people, generating renewed interest in novel eradication regimens and targets [8]. The identified property of CGNC to eradicate* H. pylori* infection can have a significant clinical importance. Some phytosubstances have a capacity to suppress* H. pylori* [10]. However, phytotherapy has not been used in clinical practice for the eradication of* H. pylori*. CGNC could be used for increasing the effectiveness of the standard* H. pylori* eradication therapy, treatment of* H. pylori* infections that have developed resistance to antibiotics, and the prevention of recurrence of the infection after the standard treatment. It seems that pathogenic microorganisms do not develop resistance to the phytoncidal multicomponent complex, CGNC, which eradicates* H. pylori*. It is important to conduct specific clinical trials using more tests for* H. pylori* diagnosis in order to obtain convincing proof of the ability of CGNC to eradicate* H. pylori*. This pilot study showed eradication of* H. pylori* in 57.1% of patients with precancerous gastric lesions using the Lesmin formulation with a lower concentration of CGNC. Given the limitations of this small pilot study, we are encouraged that more comprehensive studies of patients with* H. pylori* (with and without concomitant gastric lesions) are justified using the more concentrated capsule form of CGNC known as Bioeffective A.

Chronic inflammation and free radical damage are playing a significant role in the pathogenesis of gastritis including atrophic gastritis. Clinical investigation of patients with chronic nonatrophic and atrophic gastritis revealed the accumulation of potentially mutagenic and carcinogenic oxidant damage of DNA in cells of the gastric mucosa [50]. Suppression of inflammation and lipid peroxidation has a positive effect on the course of chronic nonatrophic and atrophic gastritis. A synergistic effect of chlorophyll, carotenoids, vitamin E, phytosterols, and other active ingredients contained in CGNC possesses strong anti-inflammatory and antioxidant properties which reduce lymphoplasmacytic infiltration of the gastric mucosa and as a result improves the morphological and functional condition of the stomach in patients with atrophic gastritis.

Several clinical interventional trials of antioxidants contained in CGNC were conducted in patients with precancerous conditions of the stomach. In Columbia, a country with a high incidence of stomach cancer, a randomised placebo-controlled study of antioxidants, used for the treatment of precancerous gastric lesions, was conducted. Use of *β*-carotene for 3–6 years led to regression of multifocal atrophy and/or intestinal metaplasia [51]. Use of vitamin E led to the regression of intestinal metaplasia in the gastric mucosa in patients with atrophic gastritis [52].

## 5. Conclusion

In summary, the results from this observational pilot study suggest that it would be feasible and important to conduct a larger randomised-controlled trial with CGNC in patients with atrophic gastritis to examine treatment of precancerous lesions and secondary prevention of stomach cancer.

## Figures and Tables

**Table 1:** Characteristics of patients before treatment.

Parameters	Group of patients	
	Treatment with CGNC	Control
Number of patients (*n*)	26	24
Women (*n*)	19	18
Men (*n*)	7	6
Average age, years, mean ± standard deviation of the mean	59.9 ± 2.4	60.7 ± 2.2
Symptoms of dyspepsia, *n* (% of patients)	26 (100%)	24 (100%)
Endoscopic signs of atrophic gastritis, *n* (% of patients)	26 (100%)	24 (100%)
Antrum: chronic atrophic gastritis, *n* (% of patients)	23 (88.5%)	21 (87.5%)
Antrum and corpus chronic atrophic gastritis, *n* (% of patients)	3 (11.5%)	3 (12.5%)
Gastric adenomatous polyps, *n* (% of patients)	7 (26.9%)	7 (29.2%)
*H. pylori* infection, *n* (% of patients)	14 (53.8%)	13 (54.2%)
Increased gastric juice pH, *n* (% of patients)	24 (92.3%)	22 (91.7%)
Decreased pepsinogen-pepsin activity in gastric juice, *n* (% of patients)	23 (88.5%)	22 (91.7%)
Decreased pepsinogen-pepsin activity in gastric mucosa, *n* (% of patients)	20 (76.9%)	20 (83.3%)
Atrophy of gastric mucosa, *n* (% of patients)		
All degrees	26 (100%)	24 (100%)
Mild	17 (65.4%)	15 (62.5%)
Moderate	8 (30.8%)	7 (29.2%)
Severe	1 (3.8%)	2 (8.3%)
Intestinal metaplasia at histological and cytological examinations, *n* (% of patients)		
All grades	16 (61.5%)	17 (70.8%)
Degree 1	7 (43.7%)	7 (41.2%)
Degree 2	6 (37.5%)	8 (47.1%)
Degree 3	3 (18.7%)	2 (11.8%)
Dysplasia	3 (11.5%)	3 (12.5%)
Lymphoplasmacytic infiltration at histological and cytological examination, *n* (% of patients)		
All degrees	26 (100%)	24 (100%)
Degree 1	12 (46.2%)	12 (50%)
Degree 2	6 (23.1%)	3 (12.5%)
Degree 3	8 (30.8%)	9 (37.5%)

**Table 2:** Symptoms of dyspepsia and endoscopic signs of gastritis in patients after six months in the treatment (600 mg per day CGNC) and control groups.

Parameters	Group of patients	
	CGNC	Control
Number of patients (*n*)	26	24
Symptoms of dyspepsia, *n* (% of patients)		
Regression	24 (92.3%)^*∗*^	3 (12.5%)
No shifts	2 (7.7%)^*∗*^	14 (58.3%)
Progression	0^*∗*^	7 (29.2%)
Endoscopic signs of gastritis, *n* (% of patients)		
Regression	24 (92.3%)^*∗*^	4 (16.7%)
No shifts	2 (7.7%)^*∗*^	16 (66.7%)
Progression	0^*∗*^	4 (16.7%)

^*∗*^Statistically significant difference between CGNC and control groups, *p* < 0.05–0.001.

**Table 3:** Detection of *H. pylori* infection in patients with precancerous gastric lesions after six months in the treatment (600 mg per day CGNC) and control groups.

Parameters	Group of patients	
	CGNC	Control
Number of patients with *H. pylori* infection before treatment (*n*)	14	13
*H. pylori *detection by rapid urease test and microscopic identification after 6 months, *n* (% of patients)		
No detection	8 (57.1%)^*∗*^	2 (15.4%)
Detection	6 (42.9%)^*∗*^	11 (84.6%)

^*∗*^Statistically significant difference between CGNC and control groups, *p* < 0.05.

**Table 4:** Functional activity of the stomach in patients after six months in the treatment (600 mg per day CGNC) and control groups.

Parameters	Group of patients	
	CGNC	Control
Number of patients	26	24
Gastric juice pH, *n* (% of patients)		
Decreasing	8 (30.8%)	4 (16.7%)
No shifts	14 (53.8%)	9 (37.5%)
Increasing	4 (15.4%)^*∗*^	11 (45.8%)
Pepsinogen-pepsin activity in gastric juice, *n* (% of patients)		
Increasing	15 (57.7%)^*∗*^	7 (29.2%)
No shifts	7 (26.9%)^*∗*^	13 (54.2%)
Decreasing	4 (15.4%)	4 (16.7%)

^*∗*^Statistically significant difference between CGNC and control groups, *p* < 0.05.

**Table 5:** Morphological features of the stomach mucosa in patients in the treatment (600 mg per day CGNC) and control groups after six months.

Parameters	Group of patients	
	CGNC	Control
Number of patients (*n*)	26	24
Intestinal metaplasia at histological and cytological examinations, *n* (% of patients)		
Regression	12 (46.2%)^*∗*^	4 (16.7%)
No shifts	9 (34.6%)	12 (50%)
Progression	5 (19.2%)	8 (33.3%)
Lymphoplasmacytic infiltration at histological and cytological examination, *n* (% of patients)		
Decreasing	14 (53.8%)^*∗*^	5 (20.8%)
No shifts	8 (30.8%)	13 (54.2%)
Increasing	4 (15.4%)	6 (25%)

^*∗*^Statistically significant difference between CGNC and control groups, *p* < 0.05.
